# Ginsenoside Rg1 Ameliorates Neuroinflammation *via* Suppression of Connexin43 Ubiquitination to Attenuate Depression

**DOI:** 10.3389/fphar.2021.709019

**Published:** 2021-08-05

**Authors:** Huiqin Wang, Yantao Yang, Songwei Yang, Siyu Ren, Juling Feng, Yangbo Liu, Haodong Chen, Naihong Chen

**Affiliations:** ^1^ Hunan University of Chinese Medicine and Hunan Engineering Technology Center of Standardization and Function of Chinese Herbal Decoction Pieces, Changsha, China; ^2^ State Key Laboratory of Bioactive Substances and Functions of Natural Medicines, Institute of Materia Medica and Neuroscience Center, Chinese Academy of Medical Sciences and Peking Union Medical College, Beijing, China

**Keywords:** depression, inflammation, ginsenoside Rg1, connexin 43, ubiquitination

## Abstract

Depression is an inflammation-associated disease that results in major depression as inflammation increases and progresses. Ginsenoside Rg1 (Rg1), the major bioactive ingredient derived from ginseng, possesses remarkable anti-depressant and anti-inflammatory effects. Our previous studies showed that the pathogenesis of depression was concomitant with the acceleration of connexin43 (Cx43) ubiquitin degradation, while Rg1 could upregulate Cx43 expression to attenuate depression. However, whether the ubiquitination of Cx43 is the specific correlation between depression and inflammation, and how Rg1 ameliorates neuroinflammation to attenuate depression, are still under investigation. In *in vivo* experiments, Rg1 treatment significantly ameliorated depression-like behaviors in rats subjected to chronic unpredictable stress (CUS). Moreover, these CUS rats treated with Rg1 exhibited attenuated neuroinflammation, together with the suppression of Cx43 ubiquitination. In *in vitro* experiments, Rg1 reduced the secretion of inflammatory cytokines and the ubiquitination of Cx43 in lipopolysaccharide-induced glial cells. Furthermore, treatment with ubiquitin-proteasome inhibitor MG132 suppressing the ubiquitination of Cx43 ameliorated lipopolysaccharide-induced neuroinflammation. The results suggest that Rg1 attenuates depression-like behavioral performances in CUS-exposed rats; and the main mechanism of the antidepressant-like effects of Rg1 appears to involve protection against neuroinflammation via suppression of Cx43 ubiquitination. In conclusion, Rg1 could ameliorate neuroinflammation via suppression of Cx43 ubiquitination to attenuate depression, which represents the perspective of an innovative therapy of Rg1 in the treatment of inflammation-associated depression.

## Introduction

Depression and inflammation are complicatedly intertwined, reinforcing and interdependent on each other. Accumulating evidence supported that inflammation is connected with the pathogenesis of neuropsychiatric diseases such as depression (Kiecolt-Glaser et al., 2015; Beurel et al., 2020). The bidirectional connection between depression and inflammation, in which depression primes larger inflammatory responses and inflammation promotes the onset of depression in turn, induces adverse health consequences to individuals and imposes massive economic burdens on both families and society (Malhi and Mann, 2018; Cruz-Pereira et al., 2020; Wang et al., 2021). The brain is a highly distinguished, heterogeneous, and intricate organ, which is responsible for maintaining the homeostasis in the central nervous system (CNS), and protecting the body from damage to the immune and nervous systems. It requires various control mechanisms, including intercellular communication through connexin (Cx)-based gap junction channels (De Sousa et al., 1993; Yuan et al., 2019). Glial cells, especially microglia and astrocytes, which ubiquitously exist in the brain (Freeman, 2010), facilitate the communication between adjacent cells through Cx-based gap junction channels and are responsible for the regulation of neuroinflammation (Freeman et al., 2017; Roy et al., 2017). Despite 21 Cx isoforms have been revealed in the human genome, Cx43 is the most prominent gap junction protein in the CNS, maintaining CNS network homeostasis, and participating in the CNS pathologies such as depression and inflammation progression (Danesh-Meyer et al., 2016; Lagos-Cabré et al., 2020). Emerging evidence has suggested that dysfunction of Cx43 is predisposed to depression (Sun et al., 2012; Xia et al., 2018), and the inflammatory reaction relies, at least in part, on intercellular communication mediated by Cx43 proteins and their channels (Willebrords et al., 2016; Medina-Ceja et al., 2019; Li et al., 2020a). Thus far, Cx43 has recently become a molecule of increasing interest for the pathogenesis and management of depression and inflammation.

The root of *Panax ginseng* C.A. Meyer (ginseng) is frequently used as a traditional herbal medicine, attributed to its numerous biological potencies against oxidation, inflammation, tumor, and depression, etc., (Lee et al., 2015; Jin et al., 2019). Ginsenoside Rg1 (Rg1), one of the most abundant and active constituents extracted from ginseng, possesses potent neuroprotective properties on anti-depression and anti-inflammation (Jiang et al., 2020). It has been extensively investigated for its anti-depressant and anti-inflammatory potentials over the past two decades (Wang et al., 2019). Our previous results indicated that the acceleration of Cx43 ubiquitin degradation was involved in the onset of depression (Xia et al., 2018), while Rg1 could upregulate Cx43 expression to attenuate depression (Xia et al., 2017). However, whether the ubiquitination of Cx43 is the correlation between depression and inflammation, and the molecular mechanism of Rg1 on anti-depression and anti-inflammation remain largely undetermined. According to the previous reports, Rg1 could rescue stress-induced depression-like behaviors *via* inhibition of inflammation (Li et al., 2020b). Multiple studies demonstrated that inhibiting the degradation of Cx43 promoted glial cells translating from a pro-inflammatory to an anti-inflammatory status (Huang et al., 2019; Wang et al., 2020). Besides, Cx43 degradation during stress requires its ubiquitination (Xia et al., 2018). Accordingly, we hypothesized that Rg1 could ameliorate inflammation via suppression of Cx43 ubiquitination to attenuate depression. In this study, we concentrated on the latent curative effect and the underlying neuroimmune mechanisms of Rg1 against depression *in vivo* and *in vitro*. Chronic unpredictable stress (CUS) is an internationally recognized method for modeling depression-like behaviors in rats (Wohleb et al., 2018; Cai et al., 2020), concomitant with undesirable inflammatory responses (Zhou et al., 2019). Additionally, lipopolysaccharide (LPS)-induced inflammation has been widely used as a model *in vitro* (Harland et al., 2020). We established models of CUS and LPS *in vivo* in rats and *in vitro* in glial cells, respectively. Then, we turned to the question of how this neuroimmune basis link existed in depression-inflammation, with a focus on the ubiquitination of Cx43. The study investigated the neuroimmune mechanism of Rg1 against depression and the role of ubiquitination of Cx43 in depression-inflammation relationships. The results might provide a novel therapy for depression with inflammation.

## Materials and Methods

### Reagents

Rg1 (PubChem CID: 441923, C_42_H_72_O_14_, ≥ 98%) was purchased from Jilin University (Changchun, Jilin Province, China). Lucifer Yellow was obtained from Sigma-Aldrich (St. Louis, MO, United States). High-glucose Dulbecco’s Modified Eagle Media (DMEM) and fetal bovine serum were produced by Gibco (Grand Island, NY, United States). Additional antibody sources: mouse *β*-actin, normal rabbit IgG, rabbit Cx43, mouse Cx43, rabbit ubiquitin, and mouse ubiquitin antibodies were obtained from Cell Signaling Technology (Beverly, MA, United States); Alexa Fluor-488 donkey anti-mouse and 546 donkey anti-rabbit secondary antibodies were produced by Invitrogen (Carlsbad, CA, United States). Protein A/G Plus-Agarose was obtained from Santa Cruz Biotechnology (Santa Cruz, CA, United States). Hoechst 33342 was produced by Dojindo Laboratories (Kumamoto, Japan). Rat and cell supernatant enzyme-linked immunosorbent assay (ELISA) Kits for interleukin-1β (IL-1β), tumor necrosis factor-α (TNF-α), caspase-1, IL-2, IL-6, and IL-18 were purchased from Abcam (Cambridge, United Kingdom).

### Animals

For animals, adult male Wistar rats weighing 250–300 g (SPF grade; purchased from Vital River Laboratories, Beijing, China) were kept at a favorable temperature of 22 ± 2°C with suitable humidity (40–60%). Rats were housed three per standard polypropylene cage and *ad libitum* access to food and water except when they are exposed to food or water deprivation during the CUS procedure. The rats were accustomed to the animal facilities for 7 days, and the sucrose baseline test was performed prior to the experiment to exclude rats that were naturally anhedonic. All experiments were performed in conformity to the National Institutes of Health Guide for the Care and Use of laboratory Animals and were approved by the Animal Care Committee of the Peking Union Medical College and Chinese Academy of Medical Sciences.

### Drug Administration and CUS to Animals

**FIGURE 1:**
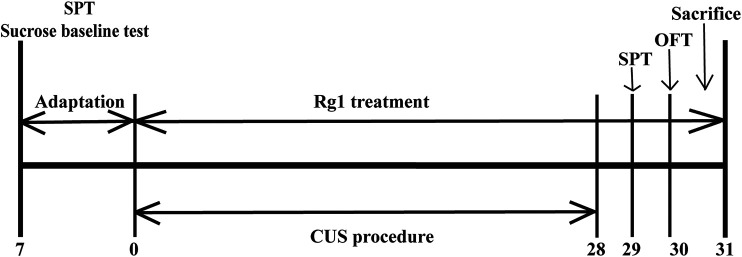
The scheme of experimental design and timeline for animals. Rats were subjected to CUS procedure in the absence or presence of Rg1 (40 mg/kg) for 28 days. Behavioral performances of rats were assessed by the SPT and OFT on days 29–30, after which the rats were sacrificed on day 31 (*n* = 6 per group). CUS, chronic unpredictable stress; Rg1, ginsenoside Rg1; SPT, sucrose preference test; OFT, open field test.

The experimental schedule applied to the animal experiments is described in Figure 1. Rats were randomly divided into three groups (*n* = 6 per group): Vehicle group, rats were administered 0.9% normal saline; CUS group, rats were administered 0.9% normal saline during CUS process; and CUS + Rg1 group, rats were administered Rg1 (40 mg/kg dissolved in 0.9% normal saline) during the CUS process. Rats in the CUS group and CUS + Rg1 group were subjected daily to the CUS procedure over 28 consecutive days, which was performed between 10:00 and 15:00, as described in Table 1. Administration by intraperitoneal injection was conducted between 8:00 and 9:00 a.m. every day, at least 1 h prior to CUS procedure. The efficacy of Rg1 on rats’ behavioral performances in the sucrose preference test (SPT) and open field test (OFT) was carried out on days 29–30 after 28 consecutive days’ stress.

**TABLE 1:** Chronic unpredictable stress procedure.

Day	Stressors
Monday	Isolation 24 h (one rat per cage); cage tilt 45°overnight
Tuesday	Tail pinch 3 min; Crowding overnight (15 rats per cage)
Wednesday	Electric shock 3 min (0.8 mA); water deprivation 12 h
Thursday	White noise 1 h (110 dB); strobe flash 2 h
Friday	Swim stress 10°C, 5 min; shaker stress 1 h (160 rpm)
Saturday	Restraint stress 4 h; reversal of the light/dark cycle (light off 12 h and light on overnight)
Sunday	Food deprivation 12 h; wet bedding overnight

### Behavioral Tests

#### SPT

The SPT was conducted as previously described (Xia et al., 2020). In brief, rats were accustomed to 1% sucrose solution for 48 h. After water deprivation for 24 h, they were exposed to two identical bottles full of either sucrose solution or water for 1 h, and the bottle positions were changed during the test to prevent the interference of bottle position on the experiment. Sucrose preference was defined as the ratio of the volume of sucrose vs total volume of sucrose and water consumed to provide an index of anhedonia.

#### OFT

The OFT was carried out as previously described (Feng et al., 2019). The bottom of the black open-field box (size: 100 cm × 100 cm × 40 cm) was divided into 25 squares equally. The high-definition camera was used to track and record the activities of each rat. During the OFT process, the total distance and number of crossing were recorded and analyzed by SMART ver. 3.0 (Panlab, S.L., Harvard Apparatus, Spain).

### Collection of Blood and Tissue Samples

Blood samples were promptly collected from abdominal aorta when the rats were anesthetized with isoflurane. Then, the serums were separated and stored at −80°C for pro-inflammation cytokines assay. Simultaneously, brains (*n* = 3) from each group were obtained by perfusion with 4% paraformaldehyde for immunofluorescence staining; brains (*n* = 3) from each group were directly decapitated to extract the cortex tissues for western blot.

### Isolation and Culture of Primary Rat Glial Cells

Primary rat glial cells were obtained from cerebral cortexes of 1-day-old Wistar rats as described previously with minor modifications (Yin et al., 2018). Isolated primary glial cells were cultivated in DMEM supplemented with 10% fetal bovine serum as well as 0.2% InvivoGen Primocin at 37°C in an appropriate atmosphere of 95% air and 5% CO_2_. After achieving confluency at around 9 days *in vitro*, the glial cells were used for the subsequent experiments.

### Inflammatory Injury to Glial Cells

Glial cells from cerebral cortexes were seeded into 6-well plates to grow up to approximately 90%. The LPS was dissolved in cell culture medium at a concentration of 1 μg/ml, as a model of inflammation *in vitro* (Cao et al., 2019). There were three groups for cell experiments: Control group, glial cells were cultivated without any treatment; LPS group, glial cells were exposed to LPS (1 μg/ml); and LPS + Rg1 group, glial cells were treated with Rg1 (1 μM) in the presence of LPS (1 μg/ml) for 24 h. After the inflammatory injury to glial cells, the supernatants from each group were collected for detecting the concentration of inflammatory cytokines. Meanwhile, the collection from LPS group was prepared as the inflammatory cytokines-conditioned medium (IC-CM). Three groups were designed to further explore the influence of inflammatory-related cytokines on gap junction intercellular communication: Control group, glial cells were cultivated with normal DMEM; IC-CM group, glial cells were treated with IC-CM; and IC-CM + Rg1 group, glial cells were treated with IC-CM supplemented with Rg1 (1 μM) for 24 h.

### Measurement of Pro-Inflammatory Cytokines

The levels of pro-inflammatory cytokines, such as IL-1β, TNF-α, caspase-1, IL-2, IL-6, and IL-18 were detected in both serum from rats and supernatant of cultured primary glial cells by their corresponding ELISA Kits, according to the protocol instructions.

### Immunofluorescence

#### Paraffin Section

After being dewaxed, permeabilized, and blocked, paraffin sections of the prefrontal cortex were incubated with a mixture of mouse anti-Cx43(1:100) and rabbit anti-ubiquitin (1:100) antibodies for 2 h at room temperature. After that, cells were conjugated with secondary antibodies of Alexa Fluor-488 donkey anti-mouse and 546 donkey anti-rabbit (1:500) for 1.5 h in the dark, and the nuclei were labeled with Hoechst 33342 (1:1,000) before being imaged by a Leica confocal microscope (Leica, Wetzlar, Germany).

#### Immunocytofluorescence Staining

After being rinsed, fixed, and blocked, cells were co-incubated with Cx43 and ubiquitin primary antibodies for 2 h and conjugated with secondary antibodies for 1 h at room temperature, the cells were incubated with Hoechst 33342 for 10 min. Images were performed using the Leica confocal microscope.

### Co-Immunoprecipitation

All procedures of Co-IP assay were performed at 4°C as described previously (Liao et al., 2013). Equal amounts (500 μg) of total extracted protein and 1 μg of rabbit antibody against Cx43 or normal rabbit IgG (Cell Signaling Technology) were mixed for 1 h. After incubation with 20 μl of Protein A/G Plus-Agarose for 2 h, the immune complexes were sedimented, washed, and resuspended in 40 μl of electrophoresis loading buffer for western blot.

### Western Blot

After the quantification of extracted proteins’ concentrations, equal amounts (30 μg) of protein sample were electrophoresed on 4–20% GLASS gels (Absin, Shanghai, China), and then transferred to polyvinylidene fluoride membranes and incubated for 2 h at room temperature with the following primary antibodies: mouse anti-Cx43 (1:1,000), mouse anti-ubiquitin (1:1,000), or mouse anti-β-actin (1:10,000). The membranes were thoroughly washed and then incubated with secondary antibody of goat anti-mouse for 1.5 h. Protein bands were performed with Image Quant LAS 4,000 mini (Sweden), and analyzed with Gel-Pro analyzer ver. 3.1 (Media Cybernetics, Bethesda, MD, United States).

### Scrape-Loading and Dye Transfer

To further evaluate the influence of inflammatory cytokines on gap junction intercellular communication, an index of depression degree, the SLDT assay was conducted as described previously with modifications (Xia et al., 2018). Briefly, after the treatment with IC-CM in the absence or presence of Rg1, cells were scraped and incubated with 0.1% (w/v) Lucifer Yellow for 6 min away from light. Images were performed by fluorescence microscopy.

### Statistical Analysis

All experimental data are represented by the mean ± SEM (bars) for three to six independent repetitions. Statistical analysis and graphing were performed with GraphPad Prism ver. 7.0 (GraphPad Software, San Diego, CA, United States). One-way analysis of variance (ANOVA) followed by Tukey’s *post hoc* test were applied to evaluate the differences among multiple groups, and an unpaired *t*-test was utilized to assess the difference between two groups. A criterion level of *p* < 0.05 was considered to determine significance.

## Results

### Rg1 Attenuates Depression-Like Behaviors in CUS-Treated Rats

**FIGURE 2:**
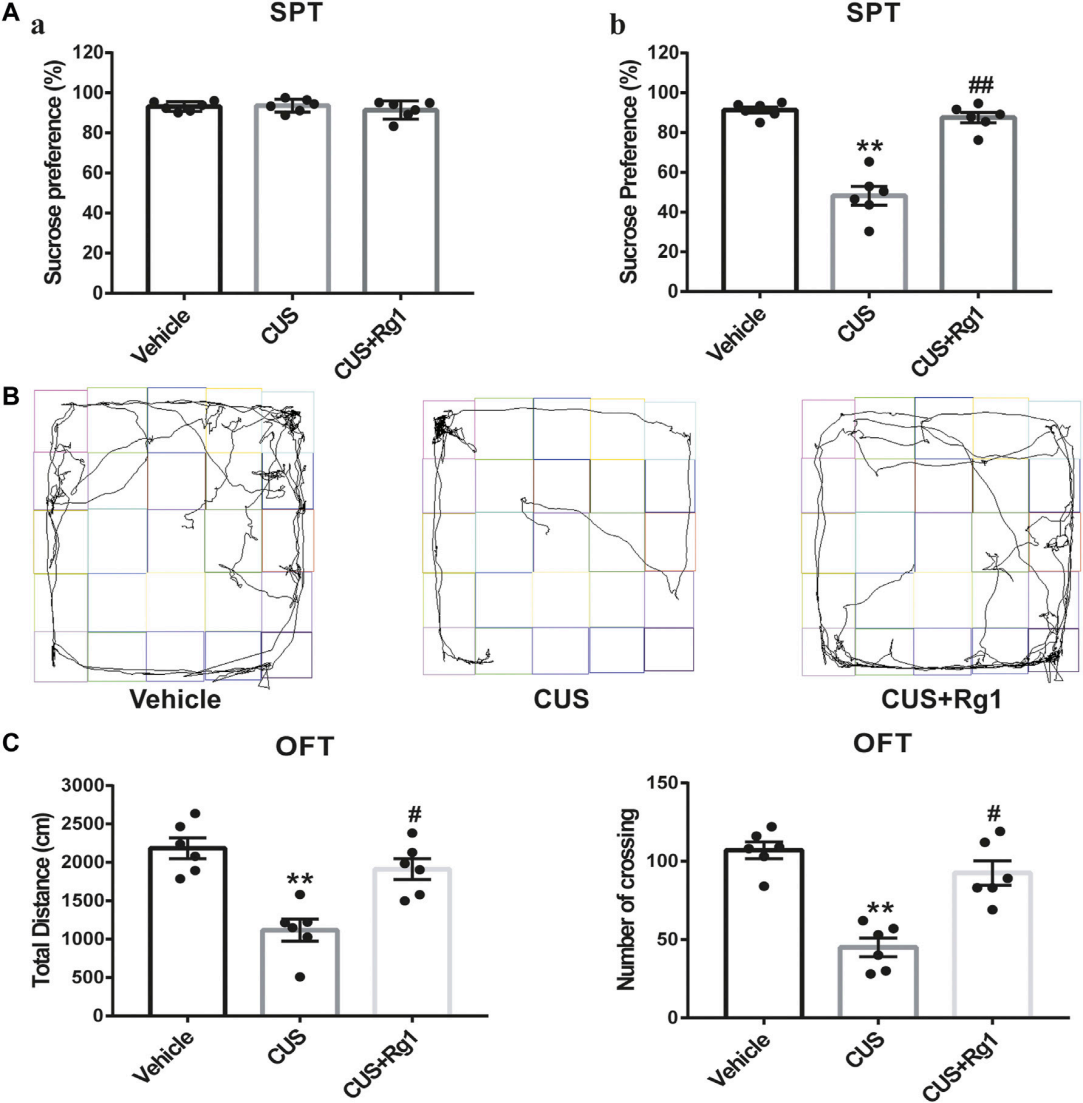
Rg1 attenuates depression-like behaviors induced by CUS exposure in rats. **(A)** The sucrose preference of rats was similar to each group before the CUS procedure (a); treatment with Rg1 prevented the decreased sucrose preference in CUS rats (b). **(B)** Representative images of the route traveled of rats in each group. **(C)** Rg1 treatment reversed the decreases in total distance and number of crossing of CUS-exposed rats in the OFT. One-way ANOVA followed by Tukey’s multiple comparisons test. All values are represented as mean ± SEM (*n* = 6). **p < 0.01, compared with the vehicle group; ^##^ *p* < 0.01 and ^#^ *p* < 0.05, compared with the CUS group.

To identify the anti-depressant effects of Rg1, we conducted the behavioral experiments of SPT and OFT. The sucrose preference was similar to each group before the CUS (Figure 2A). After the CUS, the sucrose consumption of rats was lower as compared with that of the vehicle group, while treatment with Rg1 significantly prevented the anhedonia in CUS rats (Figure 2A). As for the OFT (Figures 2B,C), the total distance traveled and the number of crossings in the CUS group were seriously decreased as compared with the non-stressed vehicle group, while Rg1 effectively reversed the decrease in total distance and the number of crossings. The results of these behavioral tests indicate the successful production of a depression model in rats, and the potent anti-depressant property of Rg1 in this CUS-induced model of depression.

### Rg1 Ameliorates Inflammatory Responses in Serum of Depressed Rats

**FIGURE 3:**
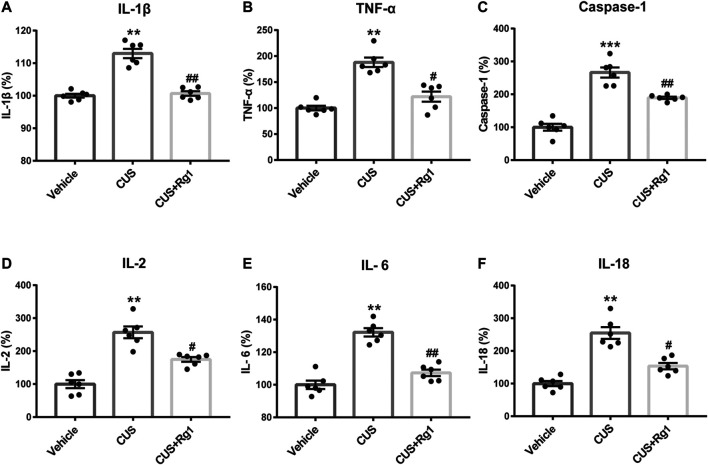
Rg1 ameliorates evaluated inflammatory responses in serum of CUS rats. Treatment with Rg1 decreased the excessive secretion of IL-1β **(A)**, TNF-α **(B)**, caspase-1 **(C)**, IL-2 **(D)**, IL-6 **(E)**, and IL-18 **(F)** caused by CUS exposure. *n* = 6 per group. **p < 0.01 and ***p < 0.001, compared with the vehicle group; ^##^ *p* < 0.01 and ^#^ *p* < 0.05, compared with the CUS group. IL, interleukin; TNF, tumor necrosis factor.

Inflammation is considered as a critical risk factor in the incidence of depression. To investigate the neuroimmune basis of the antidepressant-like properties of Rg1 in CUS-treated rats, the expression of several critical inflammatory cytokines in rat serum were examined to assess the possible involvement of neuroinflammatory responses in depression (Figure 3). The measurements of rat inflammation-related cytokines, such as IL-1β, TNF-α, caspase-1, IL-2, IL-6, and IL-18, indicated that CUS induced excessive concentrations of pro-inflammatory cytokines, compared with those assessed in the non-stressed vehicle group. However, Rg1 could significantly decrease the increases of pro-inflammatory cytokines caused by CUS exposure. Therefore, depression-like behaviors are accompanied by evaluated inflammatory responses, while Rg1 ameliorates inflammation in CUS rats.

### Rg1 Suppresses Ubiquitination of Cx43 in CUS Rats With Inflammation

**FIGURE 4:**
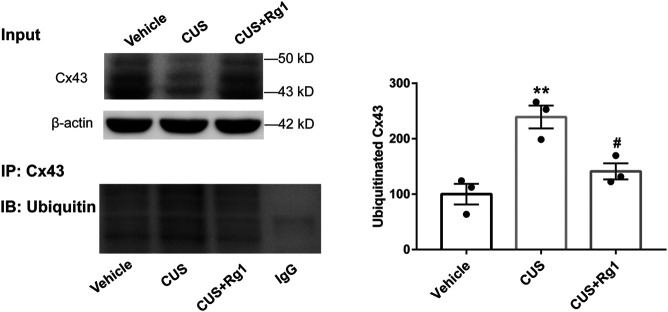
Rg1 decreases ubiquitination of Cx43 in rats exposed to CUS. The tissue lysates immunopreciptated using normal rabbit IgG or rabbit antibody against total Cx43 (IP: Cx43). The immune complexes immunoblotted using an antibody against ubiquitin (IB: Ubiquitin). The blots from three independent experiments were subjected to densitometric analyses for ubiquitinated Cx43 and the results were expressed as the density of the bands in the test sample relative to those in the vehicle sample. *n* = 3 per group. **p < 0.01, compared with the vehicle group; ^#^ *p* < 0.05, compared with the CUS group. Cx43, connexin 43.

**FIGURE 5:**
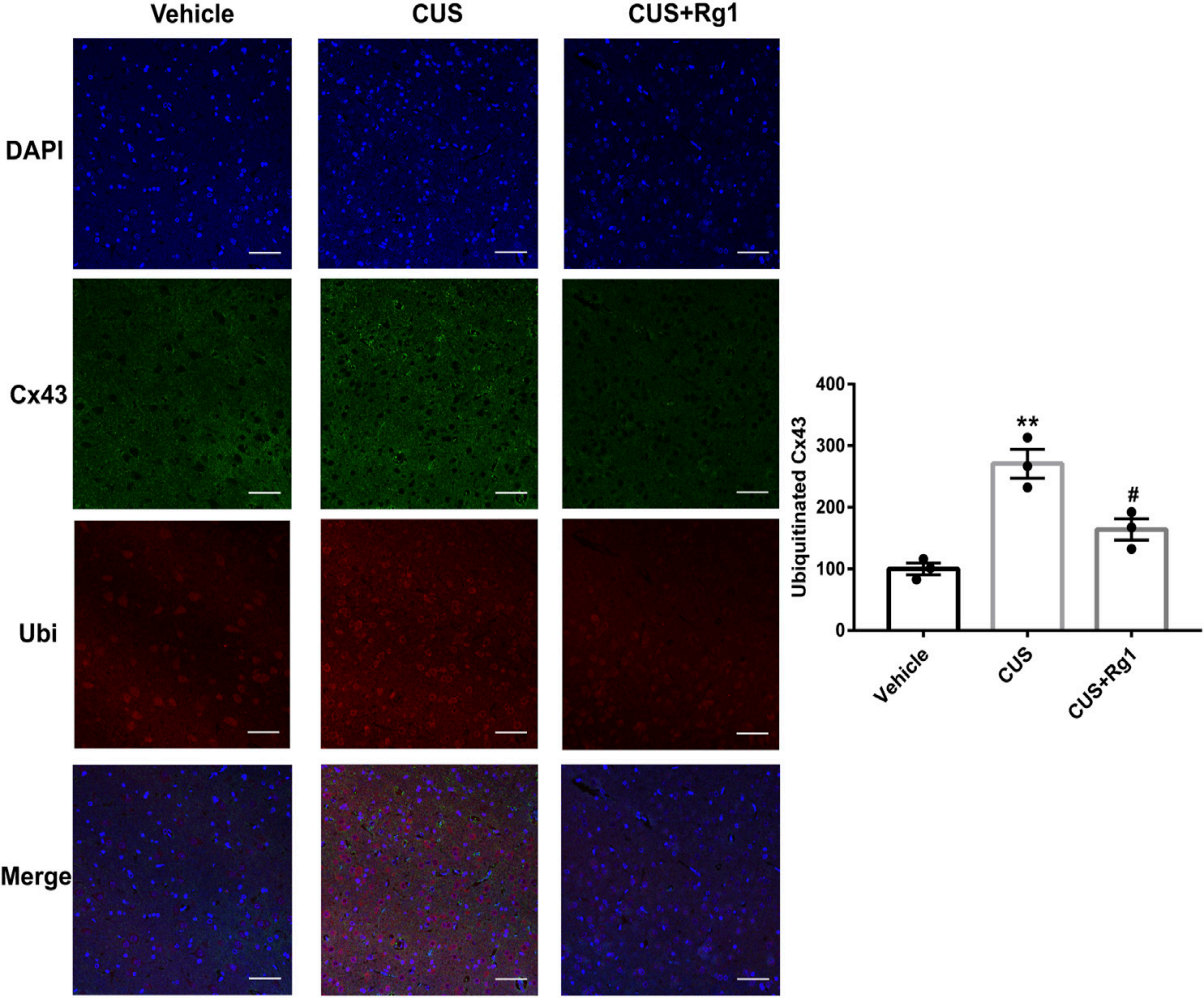
Rg1 decreases ubiquitinated Cx43 in CUS-treated rats. The confocal microscopy images showed double-stained with antibodies against Cx43 (green) and ubiquitin (red). Nuclei are stained with DAPI. Scale bar = 150 μm. *n* = 3 per group. **p < 0.01, compared with the vehicle group; ^#^ *p* < 0.05, compared with the CUS group. Ubi, ubiquitin.

Since increased activation of inflammation may result in increased ubiquitination of Cx43, and ubiquitination has been presented to be a crucial signal regulating Cx43 degradation by the proteasomal proteolytic pathway (Liao et al., 2013), we next detected the action of Rg1 on ubiquitinated Cx43 by immunoprecipitation and immunofluorescence. The upregulation of ubiquitinated Cx43 levels induced by CUS was reversed by Rg1 treatment (Figures 4, 5), indicating that the potency of Rg1 against the suppression of Cx43 ubiquitination may contribute to the alleviation of inflammation in depressed rats.

### Rg1 Reduces the Pro-Inflammatory Cytokines in Glial Cells Exposed to LPS

**FIGURE 6:**
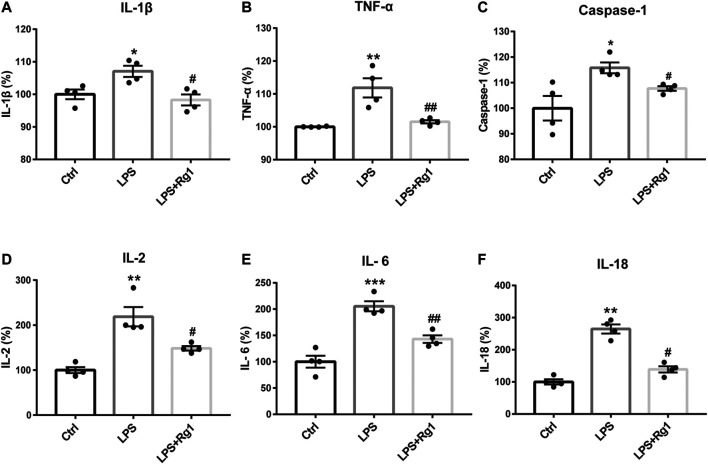
Rg1 suppresses the increased pro-inflammatory cytokines caused by LPS in glial cells. Consistent with the *in vivo* results, pro-inflammatory cytokines, including IL-1β **(A)**, TNF-α **(B)**, caspase-1 **(C)**, IL-2 **(D)**, IL-6 **(E)**, and IL-18 **(F)** were increased by LPS exposure, which was significantly suppressed by Rg1 in primary cultured glial cells. *n* = 4. *p < 0.05, **p < 0.01 and ***p < 0.001, compared with the Ctrl group; ^#^ *p* < 0.05 and ^##^ *p* < 0.01, compared with the LPS group. LPS, lipopolysaccharide; Ctrl, control.

To further explore whether Rg1 could restore neuroinflammation responses, the levels of pro-inflammatory cytokines in cell culture supernatant were measured with corresponding ELISA Kits. Consistently, LPS significantly induced the production of inflammatory cytokines in glial cells, which was significantly reduced by Rg1 to exert anti-inflammatory effect (Figure 6).

### Rg1 Ameliorates Inflammation-Induced Gap Junction Dysfunction in Glial Cells

**FIGURE 7:**
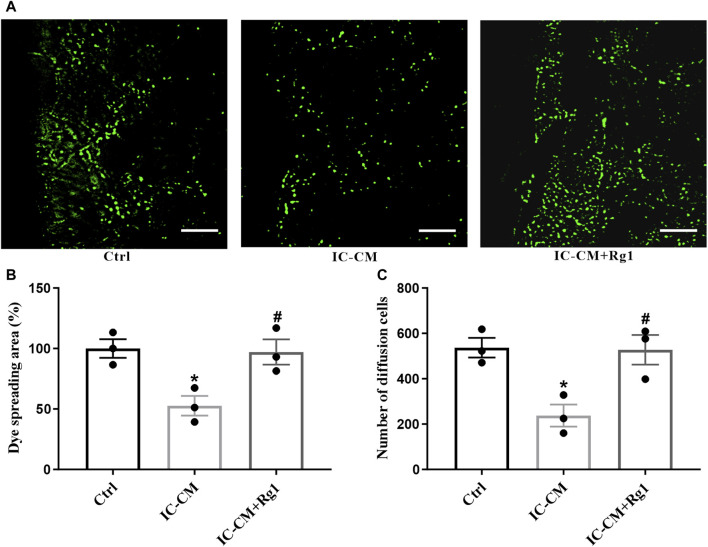
Rg1 ameliorates inflammation-induced gap junctional dysfunction in glial cells. The SLDT assay was conducted to detect the influence of Rg1 or inflammatory cytokines on gap junction intercellular communication **(A)**, which was indicated by the dye spreading area **(B)** and number of diffusion cells **(C)**. Scale bar = 100 μm. *n* = 3. ^*^ *p* < 0.05, compared with the Ctrl group; ^#^ *p* < 0.05, compared with the IC-CM group. SLDT, scrape-loading and dye transfer; IC-CM, inflammatory cytokines-conditioned medium.

Dysfunction of gap junction intercellular communication is considered as an index for depression, which can be indicated by the diffusion area of fluorescence in the SLDT assay. The IC-CM containing excessive inflammatory cytokines were applied to treat the glial cells. As shown in Figure 7, the gap junction intercellular communication in the IC-CM group was impaired, manifesting that inflammation attacked severe depression. While Rg1 could normalize inflammatory cytokines-induced reduction of diffusion area of fluorescence (Figures 7A, B) and number of diffusion cells (Figure 7C), demonstrating that Rg1 could ameliorate inflammation-induced dysfunction of gap junctions in glial cells.

### Rg1 Suppresses the Increased Cx43 Ubiquitin Degradation in LPS-Induced Glial Cells

**FIGURE 8:**
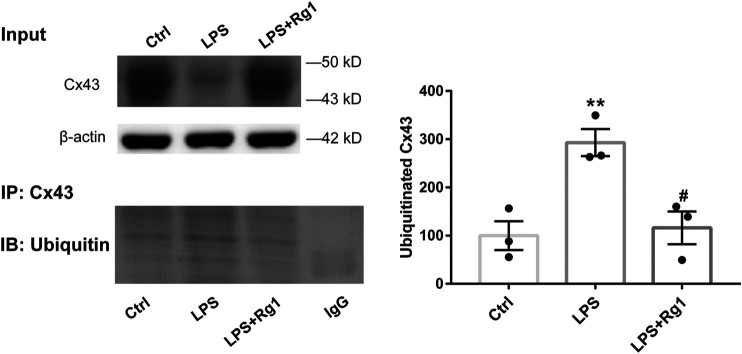
Rg1 suppresses the increased Cx43 ubiquitin degradation in LPS-induced glial cells. The cell lysates were subjected to immunoprecipitation with anti-Cx43 antibody (IP: Cx43) or normal IgG. The resultant precipitates were then subjected to western blot using anti-ubiquitin antibody (IB: Ubiquitin). Quantification of the ubiquitinated Cx43 was expressed as percentages of the control values. *n* = 3. **p < 0.01, compared with the Ctrl group; ^#^ *p* < 0.01, compared with the LPS group.

**FIGURE 9:**
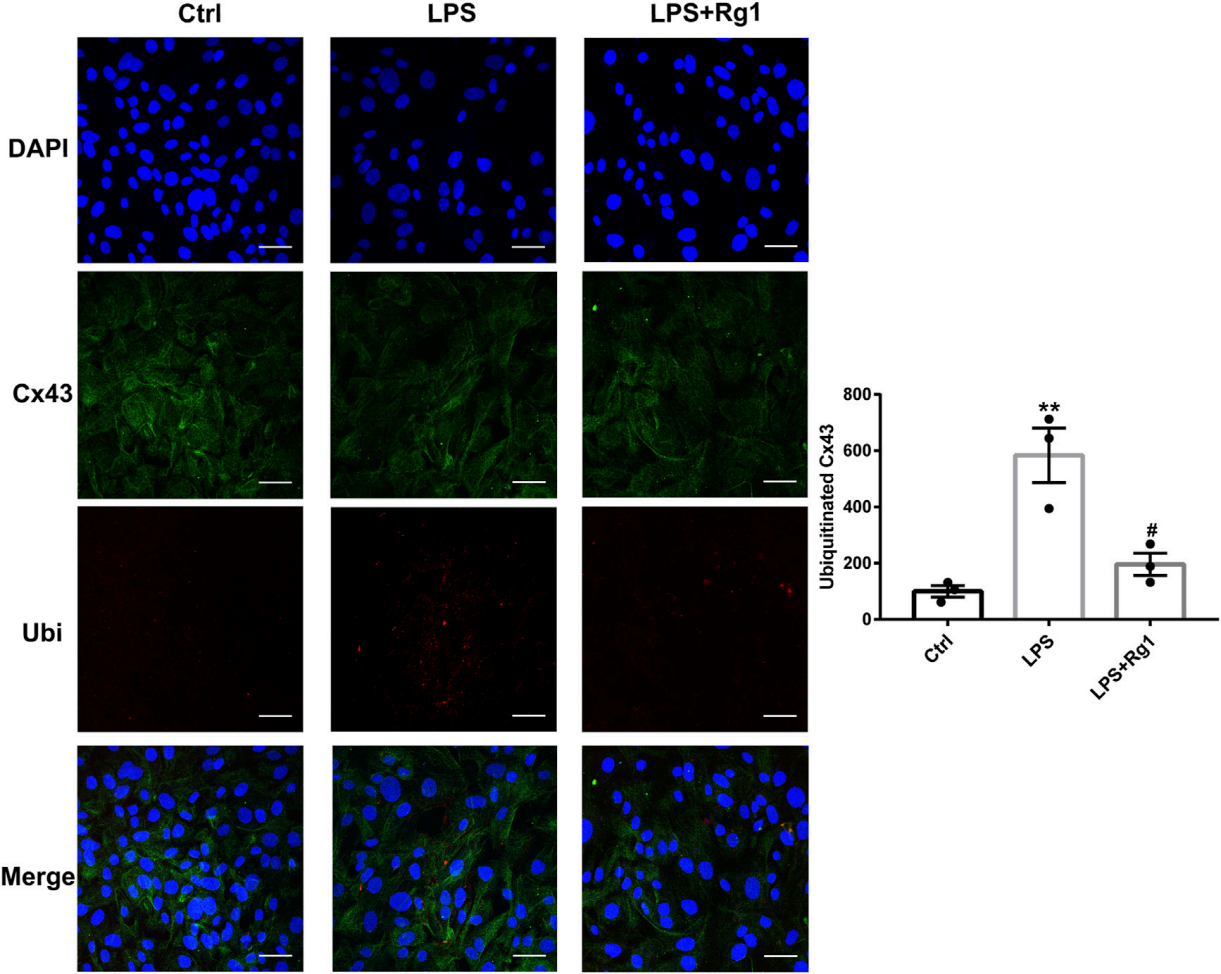
Rg1 reduces ubiquitinated Cx43 in LPS-treated glial cells. The confocal microscopy images showed double-stained with antibodies against Cx43 (green) and ubiquitin (red). Nuclei were labeled with blue. Scale bar = 150 μm. *n* = 3. **p < 0.01, compared with the Ctrl group; ^#^ *p* < 0.05, compared with the LPS group.

**FIGURE 10:**
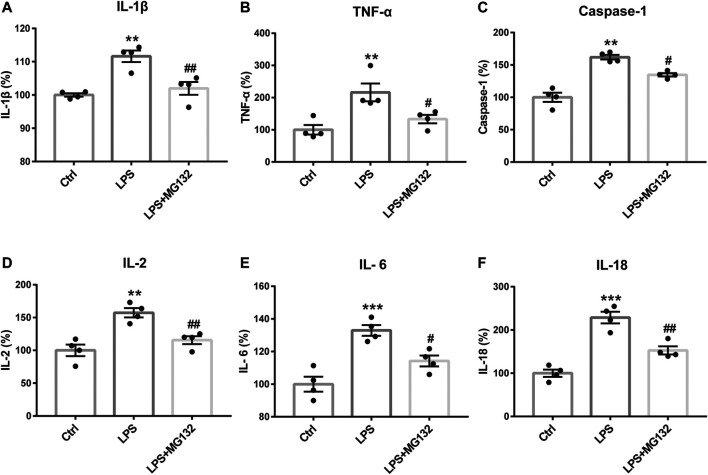
The anti-inflammatory effect of Rg1 may exert by the inhibition of ubiquitination. Inhibiting the ubiquitin-proteasome degradation by MG132 could reverse the increase of IL-1β **(A)**, TNF-α **(B)**, caspase-1 **(C)**, IL-2 **(D)**, IL-6 **(E)**, and IL-18 **(F)** caused by LPS in glial cells, which was similar to the anti-inflammatory effect of Rg1. *n* = 4. **p < 0.01 and ***p < 0.001, compared with the Ctrl group; ^##^ *p* < 0.01 and ^#^ *p* < 0.05, compared with the LPS group.

To identify the effect of ubiquitination of Cx43 in the anti-inflammatory process of Rg1, we detected the ubiquitinated Cx43 by Co-IP and immunocytofluorescence assays. As the results exhibited, LPS increased the expression of ubiquitinated Cx43 in cortical glial cells (Figures 8, 9). Rg1 reversed LPS-induced upregulation of ubiquitination of Cx43 (Figures 8, 9), indicating that Rg1 could ameliorate neuroinflammation via suppression of Cx43 ubiquitination to attenuate depression. Furthermore, we used the ubiquitin-proteasome inhibitor MG132 (10 μM) to inhibit the ubiquitination of Cx43, to investigate whether it downregulates the increased pro-inflammatory cytokines induced by LPS. Similar to the results of Rg1, MG132 reduced the pro-inflammatory cytokines compared with the LPS group (Figure 10), demonstrating that the ubiquitination of Cx43 might become a potential anti-inflammatory target. Taken together, the anti-inflammatory property of Rg1 is based on the suppression of Cx43 ubiquitination to attenuate depression.

## Discussion

In the present research, we investigated the antidepressant-like potentials of Rg1 and explored its neuroimmune basis *in vivo* and *in vitro*. In *in vivo* experiments, we applied the CUS method to generate an animal model of depression, which mimicked the chronic stressors experienced in humans. The depression-like behavioral tests such as SPT and OFT were minored to identify whether the depressive model was successfully established in rats. The results indicated that CUS treatment for 4 weeks induced robust depression-like behaviors in rats, as evidenced by the decreased consumption of sucrose solution in the SPT, as well as the reduced total distance and number of crossing in the OFT. However, treatment with Rg1 effectively reversed the deficits of depression-like behaviors, confirming that Rg1 could exert prominently anti-depressant effects in CUS-induced depressed rats.

Furthermore, the critical inflammatory factors including IL-1β, TNF-α, caspase-1, IL-2, IL-6, and IL-18 were measured to examine the action of Rg1 on inflammatory responses in CUS rats, as subsequent studies have concluded that neuroinflammation is a crucial factor recognized to be entangled in the occurrence of depression (Kohler et al., 2016). Our results maintained that CUS triggered evaluated secretion of pro-inflammatory cytokines, as assessed by the upregulation of inflammatory cytokines in the CUS group, while Rg1 treatment in depressed rats ameliorated the inflammatory reactions. The results are in line with a previous study, which revealed that Rg1 suppressed oxidative stress and neural inflammatory process in rats subjected to chronic unpredictable mild stress (Li et al., 2020b), suggesting that the antidepressant-like mechanism of Rg1 might be attributed to its anti-inflammatory property.

Cx43, the most ubiquitous Cx that contributes to the interconnection of the CNS network, is one of the more studied of the Cx isoforms, not surprisingly considering its pivotal role in normal physiology as well as its response to injury and disease. As a previous study reported, the neuroinflammation damaged Cx43-gap junction by Cx43-ubiquitin interactions (Zhang et al., 2015). Suppressing the ubiquitination of Cx43 could repair the neuroinflammatory process theoretically. It is well documented that inhibiting Cx43 degradation promoted glial cells transformed into an anti-inflammatory status (Huang et al., 2019; Wang et al., 2020). Conversely, the acceleration of Cx43 degradation triggered the inflammatory process. Besides, Cx43 degradation during stress requires its ubiquitination. The relationship between ubiquitin and Cx43 has been considered as a prominent step in the ubiquitin-proteasome pathway of Cx43 to regulate Cx43 expression and gap junction channels during inflammation processes. Accordingly, we hypothesized that Rg1 could ameliorate neuroinflammation via suppression of Cx43 ubiquitination to attenuate depression. In this study, we further elaborated the change of Cx43 ubiquitination during inflammation. Ubiquitination is a process that covalently modifies the target protein by ubiquitin, which plays a significant role in the internalization and endocytosis of Cx43 proteins (Leithe and Rivedal, 2007). Results from Co-IP and immunofluorescence staining revealed that the ubiquitination of Cx43 was involved in depression-inflammation disease, while Rg1 treatment declined the ubiquitination of Cx43 to alleviate depression and inflammation *in vivo*.

**FIGURE 11:**
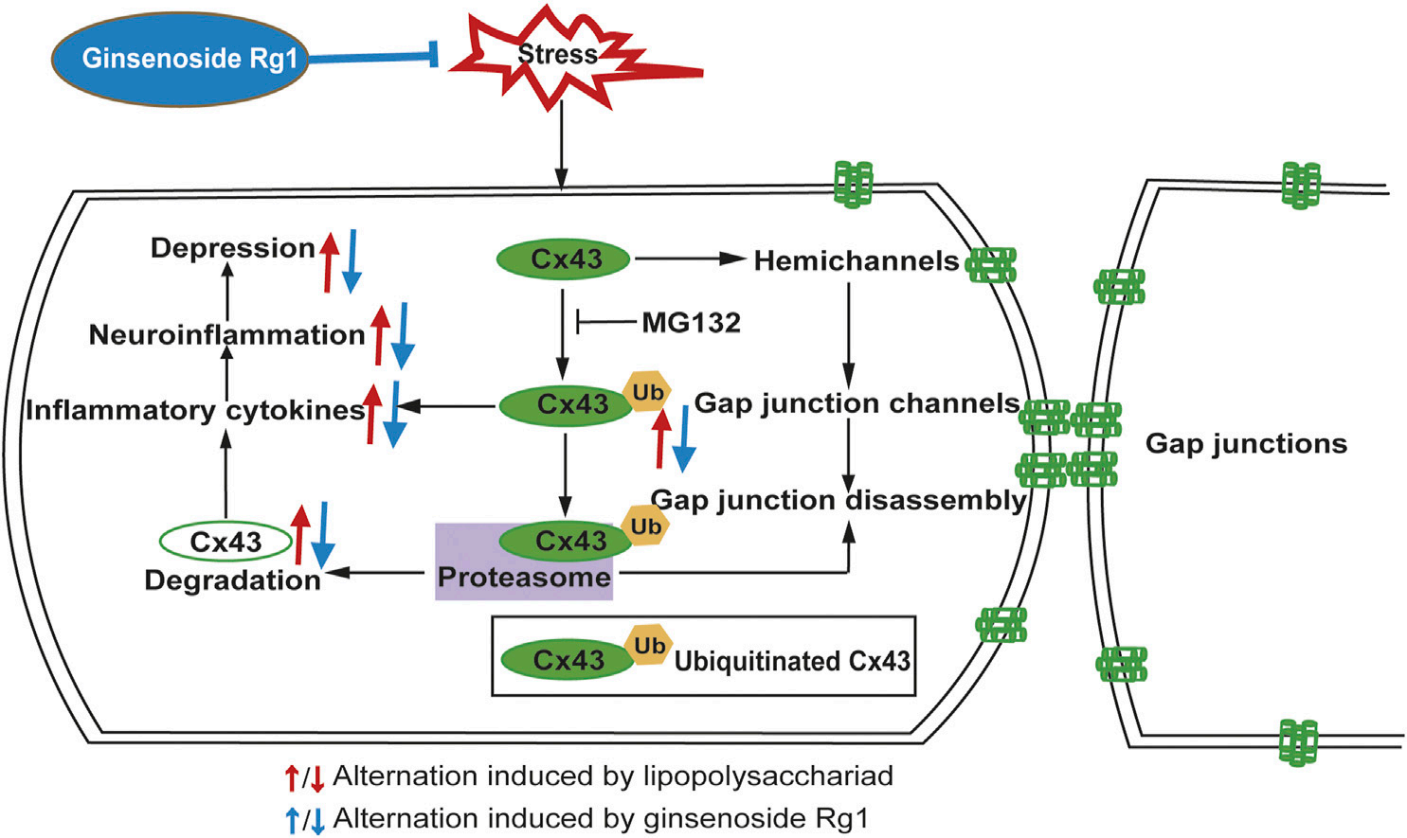
Schematic illustration of the signaling pathway involved in the anti-inflammatory and anti-depressant actions of Rg1 on Cx43 ubiquitination. Stresses, such as CUS or LPS treatment upregulates pro-inflammatory cytokines activation, which in turn aggravates depression. Rg1 may ameliorate neuroinflammation *via* suppression of Cx43 ubiquitination to attenuate depression.

LPS, which mediates the excessive release of inflammatory cytokines, is widely used to trigger an inflammatory process. In *in vitro* experiments, we applied LPS to elicit inflammatory injury to rat glial cells that play considerably crucial roles in maintaining CNS homeostasis, such as metabolic regulation of neuronal synaptogenesis, synaptic support, immune signaling, and maintenance of the blood-brain barrier (Brambilla, 2019; Bordon, 2020). The interactions between different groups of glial cell populations in inflammation is a particularly intricate and dynamic process. Our study monitored that LPS induced Cx43 ubiquitination to trigger inflammation. Moreover, Cx43 ubiquitination further promotes its autophagy, which influences the development of organisms (Levine and Klionsky, 2004), immune-mediated defense (Schmid and Münz, 2007), and prevents neuronal degeneration (Boland and Nixon, 2006). Exposure of glial cells to LPS induced a dramatic increase of pro-inflammatory cytokines and Cx43 ubiquitination, while the ubiquitin-proteasome inhibitor MG132 could downregulate the increase of pro-inflammatory cytokines, indicating that Cx43 ubiquitination is a major contribution to the neuroinflammatory responses. Taken together, Rg1 suppresses Cx43 ubiquitination to reduce the degradation of Cx43 and ameliorate neuroinflammation (Figure 11). The pharmacological targeting on Cx43 ubiquitination might represent in perspective an innovative approach to anti-depressant and anti-inflammatory therapy. However, depression is complicated, and inflammation may contribute only in a subpopulation. The intertwined links between depression and neuroinflammation need to be further investigated.

## Conclusion

In conclusion, depression-like behaviors were interrelated with inflammatory responses in a CUS-induced rat model of depression, the ubiquitination of Cx43 aggravates the inflammatory response in CUS-treated rats and LPS-treated glial cells, respectively. Rg1 may ameliorate neuroinflammation via suppression of Cx43 ubiquitination to attenuate depression.

## Data Availability

The original contributions presented in the study are included in the article/Supplementary Material, further inquiries can be directed to the corresponding author.
