# Corrigendum: Ginsenoside Rg1 ameliorates neuroinflammation via suppression of connexin43 ubiquitination to attenuate depression

**DOI:** 10.3389/fphar.2024.1418824

**Published:** 2024-07-17

**Authors:** Huiqin Wang, Yantao Yang, Songwei Yang, Siyu Ren, Juling Feng, Yangbo Liu, Haodong Chen, Naihong Chen

**Affiliations:** ^1^ Hunan University of Chinese Medicine and Hunan Engineering Technology Center of Standardization and Function of Chinese Herbal Decoction Pieces, Changsha, China; ^2^ State Key Laboratory of Bioactive Substances and Functions of Natural Medicines, Institute of Materia Medica and Neuroscience Center, Chinese Academy of Medical Sciences and Peking Union Medical College, Beijing, China

**Keywords:** depression, inflammation, ginsenoside Rg1, connexin 43, ubiquitination

In the published article, there was an error in Figure 7 as published. The images for IC-CM and IC-CM + Rg1 in panel A need to be corrected. The added panel C provides an additional statistical indicator to increase the reliability of the results. The corrected Figure 7 and its caption appear below.

**FIGURE 7:**
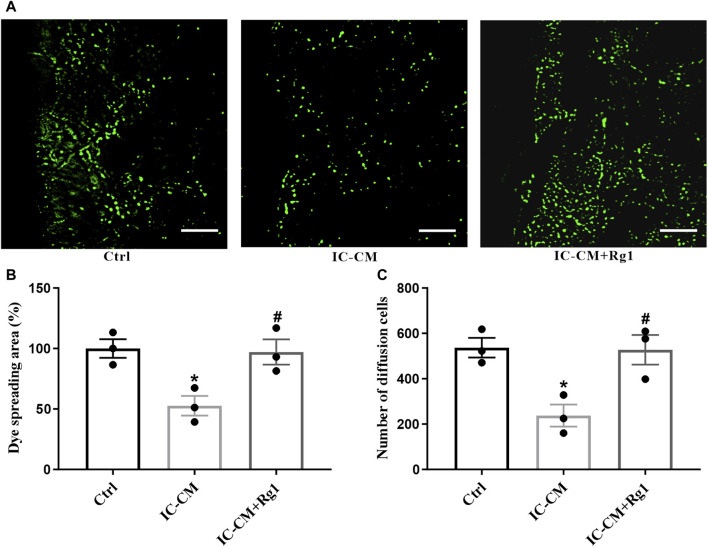
Rg1 ameliorates inflammation-induced gap junctional dysfunction in glial cells. The SLDT assay was conducted to detect the influence of Rg1 or inflammatory cytokines on gap junction intercellular communication **(A)**, which was indicated by the dye spreading area **(B)** and number of diffusion cells **(C)**. Scale bar = 100 μm. *n* = 3. ^*^ *p* < 0.05, compared with the Ctrl group; ^#^ *p* < 0.05, compared with the IC-CM group. SLDT, scrape-loading and dye transfer; IC-CM, inflammatory cytokines-conditioned medium.

In the published article, there is an update.

A correction has been made to **Results**, *Rg1 ameliorates inflammation-induced gap junction dysfunction in glial cells*, Paragraph Number 1. The two sentences previously stated:

“As shown in Figure 7, the gap junction intercellular communication in the IC-CM group was impaired, manifesting that inflammation attacked severe depression. While Rg1 could normalize inflammatory cytokines-induced reduction of diffusion area of fluorescence, demonstrating that Rg1 could ameliorate inflammation-induced dysfunction of gap junctions in glial cells.”

The corrected sentences appear below:

“As shown in Figure 7, the gap junction intercellular communication in the IC-CM group was impaired, manifesting that inflammation attacked severe depression. While Rg1 could normalize inflammatory cytokines-induced reduction of diffusion area of fluorescence (Figures 7A, B) and number of diffusion cells (Figure 7C), demonstrating that Rg1 could ameliorate inflammation-induced dysfunction of gap junctions in glial cells.”

The authors apologize for these errors and state that this does not change the scientific conclusions of the article in any way. The original article has been updated.

